# Understanding Sex-Based Kinematic and Kinetic Differences of Chasse-Step in Elite Table Tennis Athletes

**DOI:** 10.3390/bioengineering9060246

**Published:** 2022-06-04

**Authors:** Xiaoyi Yang, Qichang Mei, Shirui Shao, Wenjing Gu, Yuqi He, Ruizhe Zhu, Yaodong Gu

**Affiliations:** 1 Faculty of Sports Science, Ningbo University, Ningbo 315211, China; 2011042109@nbu.edu.cn (X.Y.); heyuqi0809@outlook.com (Y.H.); justzhe2022@163.com (R.Z.); guyaodong@hotmail.com (Y.G.); 2 Research Academy of Grand Health, Ningbo University, Ningbo 315211, China; 3 Auckland Bioengineering Institute, The University of Auckland, Auckland 1010, New Zealand; 4 Faculty of Basic Teaching, Nantong Institute of Technology, Nantong 226002, China; guwenjing0248@163.com; 5 Faculty of Engineering, University of Pannonia, H8201 Veszprém, Hungary

**Keywords:** kinematic, kinetic, table tennis, sex, footwork, lower extremity

## Abstract

With the progress and innovation of table tennis technology, individualized training programs may deserve special attention. This study aimed to analyze elite table tennis athletes in chasse-step, with a particular focus on sex-based biomechanical differences. A total of 36 (18 males and 18 females) elite table tennis athletes performed topspin forehand of chasse-step. Angles and moments of hip, knee, and ankle joints were calculated using OpenSim (v4.2) with marker trajectories and ground reaction forces were measured via Vicon motion capture system and AMTI in-ground force platform. Males had greater hip and knee flexion angles during the entire motion phase and greater internal rotation angles of the hip during the forward swing phase. The joint stiffness of knee in males was greater than females in the frontal plane. Females in the forward swing phase showed greater hip flexion, adduction, and internal rotation moments than males. It was suggested that the difference may be due to the limitation of anatomical structures in sexes. Male table tennis athletes should strengthen lower extremity muscle groups to improve performance, while female table tennis athletes should focus on hip joint groups to avoid injury. The sex differences presented in this study could help coaches and athletes to develop individualized training programs for table tennis.

## 1. Introduction

As one of the major racket sports, table tennis has been included in Olympic programs since 1988 [1]. The complexity of table tennis game lies in that the physical capability, technique and tactics, mental state, and fitness of athletes are all factors that affect the level of achievement [2,3]. Particularly, the movement techniques are the crucial factors for table tennis athletes, which consist of stroke, position, footwork, and many other skills. A high level of techniques means that table tennis athletes are able to use proper footwork, control the power, and stroke the ball with adequate speed and spin [4,5]. The analysis of table tennis techniques could help coaches and athletes identify the key factors for effective table tennis stroke.

One of the key aspects of table tennis technique is the footwork; using proper footwork techniques allows table tennis athletes to achieve the perfect position to stroke effectively [6]. Some researchers have investigations emphasized the importance of footwork in table tennis performance. By investigating the one-step, chasse-step, and cross-step of 15 male table tennis players, it was suggested that the difference of the joint loadings in the chasse-step and cross-step may provide useful insights into injury mechanisms and training program development for table tennis [7]. Yu et al. found that long chasse-step of the hip, knee, and ankle joints of short chasse-step were more likely to be injured [8]. Malagoli Lanzoni and Lobietti compared the footwork of international and national players and found that Asians used the most effective strokes and footwork more frequently than Europeans [1]. Malagoli Lanzoni et al. collected data from male and female table tennis athletes and found that usage of chasse-step was equally 20% [6].

Surprisingly, the development of table tennis athletes’ technical level requires individualized training programs. In previous studies, Bankosz and Winiarski found that the kinematic parameters of table tennis topspin forehand were highly variable between inter-and intra-individual, and concluded the necessity of personalized training programs [2]. The purpose of individualized training is to the adjust training load and program according to the different needs of athletes [9]. Individualized training programs are indispensable for optimizing sex-appropriate development techniques [10].

In fact, sex differences are reflected in many sports. McLean et al. found significant sex effects in knee, hip, and ankle joints kinematics by comparing male and female basketball athletes, and female basketball athletes were at higher risk of anterior cruciate ligament injury [11]. Other researchers suggested that there may be different biomechanical loading mechanisms between males and females by finding that the trunk and pelvic kinematics of young rowers were different during rowing [12], which was also found in young runners [11]. Nevertheless, little has been reported on the biomechanics of sex in table tennis athletes.

In particular, table tennis is an endurance-intensive sport [13]. Despite the popularity of table tennis, little is known about the characteristics required for table tennis athletes of both sexes. Due to the complexity of this sport, it is difficult for researchers to gather comprehensive information that could be used by coaches to develop individualized training programs for athletes to improve performance. The objective of this study was to investigate the effects of sex difference on kinematic and kinetic in elite table tennis athletes while performing chasse-step. It was hypothesized that there would be significant differences between male and female athletes in kinematic and kinetic during chasse-step. Individualized approaches for male and female athletes may provide a better basis for the development of training plans and perfect performance in competitions.

## 2. Materials and Methods

### 2.1. Participants

The study recruited 36 (18 males and 18 females) elite competitive table tennis athletes (National Level I). All the participants (Table 1) were recruited from in University Table Tennis Team and all the participants were right-handed, which was determined with the racket hand. The experiment was conducted in strict medical screening to ensure that the participants were physically healthy and had no muscle and joint disorders in the last six months. In addition, the participants were clearly informed of the experimental requirements and the possible problems before the experiment. Each of the participants was required to sign the informed consent form. The protocol of this study was approved by the Ethics Committee of Research Academy of Grand Health at Ningbo University (RAGH20211116).

### 2.2. Experimental Procedures

**Figure 1:**
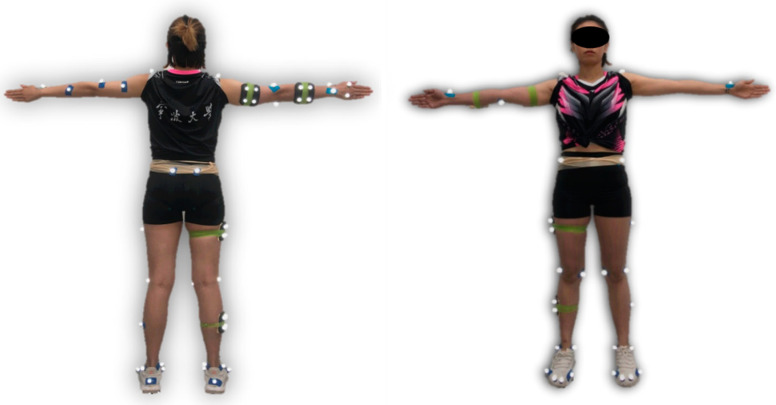
The anatomical locations of markers attached on the table tennis athlete.

Eight-camera motion capture systems (Oxford Metrics Ltd., Oxford, UK) recorded the marker trajectory at a frequency of 200 Hz. The AMTI in-ground force platform was employed to record the ground reaction force (AMTI, Watertown, MA, USA), at a frequency of 1000 Hz. The marker-set included 52 markers (diameter: 14 mm) in the test. These markers were attached to the upper and lower limbs, including left and right of shoulder, distal joint of humerus, radius and ulna, the proximal joint of the second and fifth phalanx, iliac spine, condyle, malleolus, first and fifth metatarsal heads, distal joint of the first and second toe, as well as tracking clusters attached to the elbow, wrist, thigh, shank, and heel (Figure 1).

**Figure 2:**
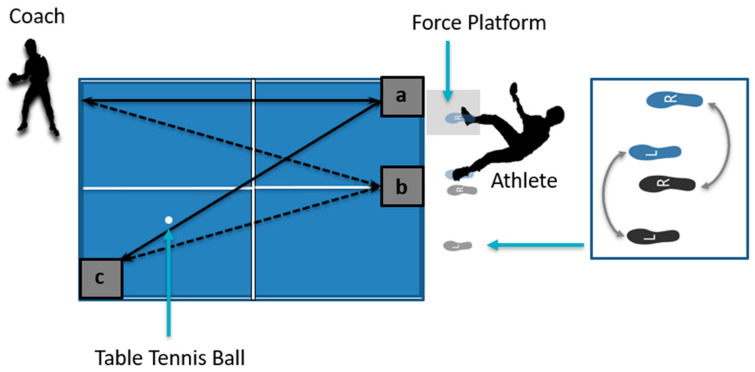
Experimental setup of First Impact Area (**a**), Second Impact Area (**b**), Target Area (**c**), and topspin forehand using chasse-step.

The experimental environment simulated the table tennis training ground and scene. The experimental stroke area consisted of two impact areas and one target area of 20 × 20 cm, traced upon a regular table tennis table which used in formal table tennis competition. All participants held the same table tennis racket to stroke. The table tennis ball used was that which was used in the national games (D40+, red doubles, Shanghai, China). Athletes used chasse-step to carry out a five-minute training session before the experiment. Each participant collected a static marker trajectory and ground response data. The professional coach served table tennis balls to athletes, requiring the chasse step to return the balls as in real-scenario competition. Five dynamic trials were recorded for each participant during data collection that were judged by the same professional coach serving balls in the test. Participants performed stroke topspin with the forehand from impact areas to the target area. The chasse-step after stroking formed the first impact area (Figure 2b, gray step) to the target area (Figure 2c), then participants slid their left lower extremity close to their right lower extremity and then stepped their right lower extremity toward the right to prepare the second topspin forehand stroke (Figure 2a, blue step) [7].

### 2.3. Data Processing

**Figure 3:**
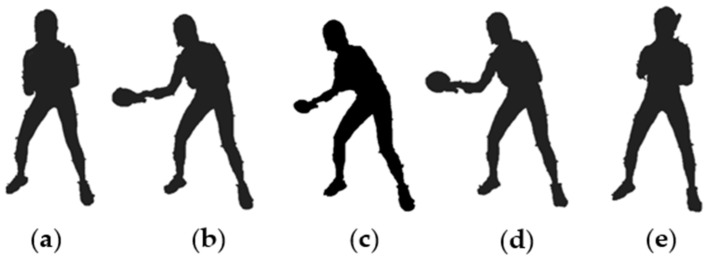
The stance interval of the chasse-step in this study, including (**a**) Ready Position, (**a**–**c**) Backward Swing Phase, (**c**) Backward Swing termination, (**c**–**e**) Forward Swing Phase, and (**e**) Forward Swing termination.

This study adopted the OpenSim model (v4.2) to process data based on the established pipeline [14]. The movement stance was divided into the part where the participant was landing on the force platform in this study. After standardization, it was found that the participants entered the backward swing phase (Figure 3b) when landing on the force platform, about 30% of the entire stance was the backward swing end (Figure 3c), followed by the forward swing phase (including the topspin forehand stroke) (Figure 3d), and about 55% (Females)–60% (Males) of the entre stance was the forward swing end (Figure 3e), before returning to the ready position (Figure 3a). The model was scaled up using the static marker position of each participant to achieve a certain model of motion matching. The angles of the lower extremity joint were calculated using the Inverse Kinematics (IK) algorithm to reduce the error and then the angles and ground reaction forces were used to compute joint moment using the Inverse Dynamic (ID) algorithm. The joint stiffness was calculated via change of joint moments (N/kg) and change of joint angles (degree).

### 2.4. Statistical Analysis

Differences between male and female table tennis athletes were assessed using an Independent *t*-test with *p* = 0.05 as a cut-off value. The normality was checked for the normal distribution of the variables with the Shapiro–Wilk test. After that, the open-source SPM 1D package was employed for statistical analysis using the MATLAB R2019a (The MathWorks, Natick, MA, USA).

## 3. Results

### 3.1. Kinematics and Kinetics

#### 3.1.1. Hip Joint

**Figure 4:**
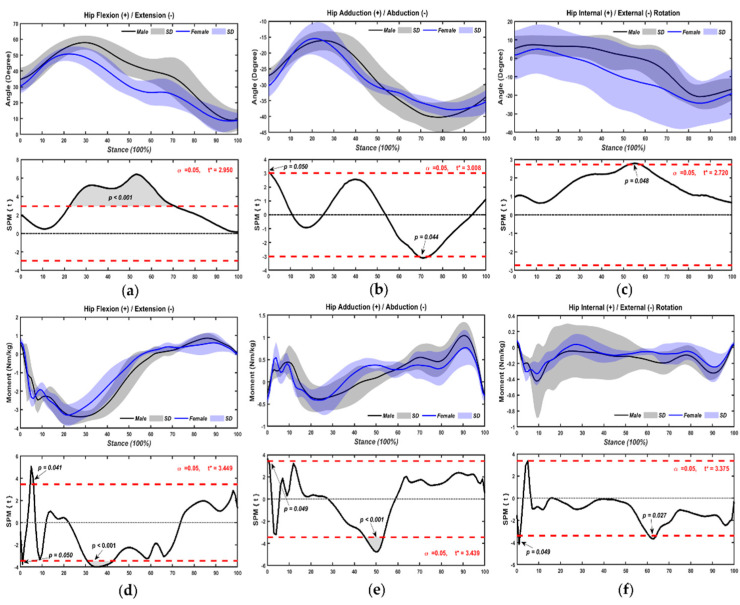
Kinematics ((**a**), flexion/extension angle; (**b**), adduction/adduction angle; (**c**), internal/external rotation angle) and kinetics ((**d**), flexion/extension moment; (**e**), adduction/abduction moment; (**f**), internal/external rotation moment) of hip joint with chasse-step in sexes.

As outlined in Figure 4, kinematics and kinetics of hip joint showed significant differences during chasse-step of table tennis. Hip joint of male table tennis athletes increased flexion at 20–70% (*p* < 0.001) (Figure 4a) during the stance. Chasse step of male athletes increased more in adduction at the first stance (*p* = 0.05) and more abduction by 70% (*p* = 0.044) (Figure 4b) than female table tennis athletes. Additionally, male table tennis athletes’ internal rotation of the hip joint increased by 55% (*p* = 0.048) (Figure 4c) during chasse-step of table tennis.

The hip flexion moment of male athletes was demonstrated as significantly greater by 4–8% (*p* = 0.041), but decreased by 2% (*p* = 0.05) and 32–43% (*p* < 0.001) (Figure 4d) relative to female athletes. Furthermore, male athletes increased the adduction moment more at the beginning of their stance, and the abduction moment more by 45–53% (*p* < 0.001) (Figure 4e) when compared with females. The external rotation moment of male athletes increased by 2–4% (*p* = 0.049), and 61–65% (*p* = 0.027) (Figure 4f) with the chasse-step stance.

This section may be divided by subheadings, which aimed to provide a concise and precise description of the experimental results, interpretation, as well as the experimental conclusions that can be drawn.

#### 3.1.2. Knee Joint

**Figure 5:**
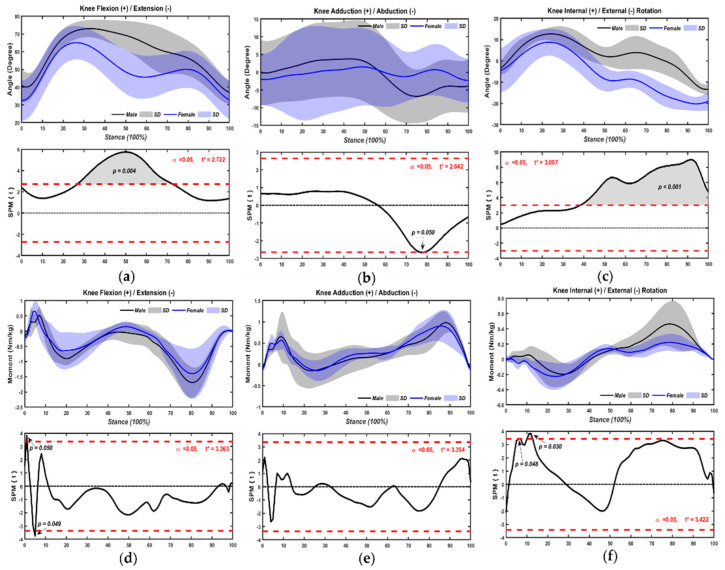
Kinematics ((**a**), flexion/extension angle; (**b**), adduction/abduction angle; (**c**), internal/external rotation angle) and kinetics ((**d**), flexion/extension moment; (**e**), adduction/abduction moment; (**f**), internal/external rotation moment) of knee joint with chasse-step in sexes.

Kinematic and kinetic deviations for the knee joint parameters were graphically represented in Figure 5. The knee flexion angles of male table tennis athletes increased more at 25–73% (*p* = 0.004) (Figure 5a) than female athletes. Additionally, males exhibited a significantly greater abduction at 78% (*p* = 0.05) (Figure 5b). For females, there was greater external rotation from 40% to the end (*p* < 0.001) (Figure 5c) during the chasse-step stance.

Male table tennis athletes tended to have slightly greater knee flexion by 1–2% (*p* = 0.05), but females tended to be at 5–6% (*p* = 0.049) (Figure 5d). No differences were found in adduction and abduction moments of knee joints between male and female table tennis athletes over time. Within-sex comparisons revealed the peak male athletes of table tennis increased their internal rotation moment by 7–8% (*p* = 0.048), 10–13% (*p* = 0.003) (Figure 5f).

#### 3.1.3. Ankle Joint

**Figure 6:**
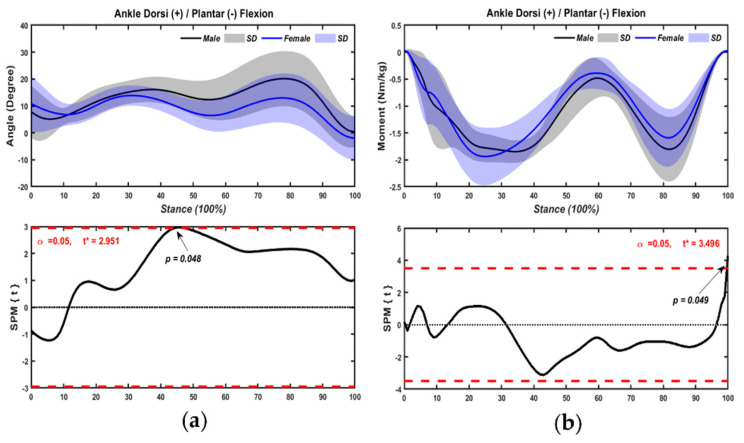
Kinematics ((**a**), dorsi/plantar flexion angle) and kinetics ((**b**), dorsi/plantar flexion moment) of ankle joint with chasse-step in sexes.

Figure 6 presented a summary of kinematic and kinetic comparisons of the variables of ankle joint for male and female table tennis athletes with chasse-step. Male athletes performed greater dorsiflexion angles by 44–48% (*p* = 0.048) (Figure 6a), and dorsiflexion moment by 99–100% (*p* = 0.049) (Figure 6b) compared with female athletes with chasse-step.

### 3.2. Stiffness of Lower Extremity

The stiffness of lower extremity joints was associated with the sex of table tennis athletes in the sagittal, frontal, and transverse planes in Table 2. Overall, the hip stiffness of males was greater than females in both sagittal and transverse planes, though the significance level was not reached. In addition, compared with females, males showed greater stiffness in all three planes at the knee joint, with only significant difference observed in the frontal plane (*p* = 0.02) (Table 2). In the sagittal plane of the ankle joint, stiffness was significantly larger in females on average (*p* = 0.006) (Table 2).

## 4. Discussion

Using proper footwork including chasse-step is a basic technique, and moving to the perfect position enabled table tennis athletes to stroke effectively and efficiently [6]. The aim of this study was to compare elite male and female table tennis with chasse-step in lower-extremity kinematics and kinetics. This study demonstrated significant sex differences in elite table tennis athletes during chasse-step. The results showed many sex differences during chasse-step. Males showed more hip and knee flexion angles over stance time of chasse-step. Compared with females, males displayed more knee internal rotation during the forward swing phase. Besides, female table tennis athletes showed more hip flexion moments in the forward swing phase than males. Greater hip adduction and internal rotation moment in females was also observed during the forward swing phase. In the frontal plane, the stiffness of the knee joint in males was significantly greater than females from statistical analysis.

Table tennis athletes not only require physical strength, but also physical control to be prepared for a quick stroke [13]. The variation in angle parameters from the backward to forward swing phase of lower extremities suggested that the chasse-steps of male table tennis athletes were more involved in the knee and hip joints. Gu et al. found that the dynamic physical control ability of male table tennis athletes was greater than females [15]. Yang et al. also agreed that male table tennis athletes had better physical control to speed up backward and forward swing phases in a shorter time, which was accelerative to an effective stroke [16]. Muscle performance aspects seemed to be essential for elite table tennis athletes in developing physical control [17]. Males showed greater stiffness in the knee joint compared with females in chasse-step. Granata et al. also proved that there were sex differences in stiffness associated with the lower extremity [18]. Muscle stiffness was shown to be proportional to muscle mass between young males and females [19]. In a study of 52 healthy young individuals, Lim and Choi found that the stiffness of males was 18% greater than females [20]. The male athletes exhibited characteristic stiffness, which was beneficial to their athletic performance [21]. Compared with females, male table tennis athletes with greater muscle masses had a larger bend in their torso and knees on the topspin forehand, which results in a maximum acceleration difference between sex [22]. It could be inferred that greater muscle masses allowed males to generate higher strength than females [23], and thus enabled male table tennis athletes to perform better than females. Perhaps it was due to different anatomical structures leading to different inherent strength biomechanics in males and females. This finding was in general agreement with other sex-based sports. The difference in kinematics of handball throwing between males and females may be caused the anatomical structures [24]. Therefore, the limitations of anatomical structures emphasize the benefits of individualized training as necessary to develop between male table tennis athletes and female table tennis athletes.

Significant differences between male and female table tennis athletes with chasse-step were noted with hip flexion, adduction, and internal rotation moment. The differences in lower extremity injury hip muscle strength of collegiate athletes between males and females reported that the incidence of lower extremity injury in female athletes was about 5% more than that in male athletes [25]. In addition, muscle imbalance was thought to result from weak muscles on one side of the joint while those on the other side were strong [26]. It was also found that female athletes with bilateral hip extension imbalance had a 15% or more injury rate in the upper and lower extremities compared with the other side [27]. Female table tennis athletes exhibited larger hip moments during forward swing phase than males. It indicated that females required their hip to exert more force to stroke, possibly inferring that injury risk to the hip joint of females is higher than in males. It is worth attention that overuse of the hip joint was associated with hip joint groups injured, for example, lower back pain, and dysfunction in the hip joint could increase pressure on other joints [28]. Not only low back pain was common, but the incidence in athletes was also up to 30% [29]. Female athletes are proven to be more prone to the low back pain than male athletes; according to the 1997–1998 NCAA Injury Monitoring data, females are almost twice as likely as males to have lower back pain [25]. Lower back pain is the most common injury in female volleyball matches which, are also the second and third most common spring injuries in basketball and football of females [30]. Comparing female college athletes who participated in nine fields which including volleyball, basketball, futsal, tennis, badminton, swimming, tracking, shooting, and Karate in National Sports Olympiad of Female University Students, Noormohammadpour et al. found that female athletes who practice basketball and karate have a higher prevalence of lower back pain than other sports [31]. Additionally, similarly to the high repeated load on the hip joint in table tennis, female athletes were found to have lower back pain in combat sports [32]. This may imply that female table tennis athletes are more prone to lower extremity injury compared with males, especially lower back pain.

As a limitation of this study, individual differences in knee abduction and adduction in male and female athletes and hip rotation in female athletes were not illustrated. It could be seen from the ankle and hip stiffness in the sagittal and frontal planes of females that perhaps these may be due to the higher level of female athletes compared with the average female table tennis players. Another limitation was that upper limbs or the whole body should be investigated and discussed as a whole-body chain, not simply the lower extremity, which shall be noted in future studies. Furthermore, table tennis athletes used a variety of footwork to move into a perfect position to stroke. Chasse-step in this study was one of the most common footwork [1], and the present findings might not be applicable to the forehand strokes in all table tennis situations.

## 5. Conclusions

The present study analyzed the kinematic and kinetic difference in the lower extremity between male and female table tennis athletes during chasse-step. The difference found in angles and moments between males and females may be the manifestation of sex differences during chasse-step. Due to the differences and limitations of anatomical structures, male athletes’ movement patterns may benefit more from the involvement of large muscle groups in the lower extremities during chasse-step. Female athletes should consider focusing on large physical sections of training to improve performance. Coaches and athletes of table tennis could strengthen individual training programs so as to improve match performance.

## Figures and Tables

**Table 1:** Participants (*n* = 36) characteristics and anthropometric measures (Mean ± SD).

Variable	Age (Years)	Height (cm)	Weight (kg)	Training Experience (Years)
Male	21 ± 1.34	175 ± 5.54	74 ± 2.79	13 ± 2.53
Female	20 ± 0.71	164 ± 1.00	54 ± 4.92	13 ± 0.99

**Table 2:** Stiffness values of lower extremity joints between sexes (Mean ± SD).

Joint	Plane	Male	Female	*p*-Value
Hip	X	0.178 ± 0.034	0.169 ± 0.021	0.06
	Y	0.071 ± 0.019	0.080 ± 0.025	0.06
	Z	0.080 ± 0.050	0.040 ± 0.026	0.07
Knee	X	0.052 ± 0.007	0.048 ± 0.012	0.48
	Y	0.258 ± 0.190 *	0.135 ± 0.046 *	**0.02**
	Z	0.026 ± 0.004	0.019 ± 0.008	0.42
Ankle	X	0.113 ± 0.028 *	0.194 ± 0.110 *	**0.0006**

* Indicates significant differences between male table tennis athletes and female athletes (*p* < 0.05); X, the sagittal plane; Y, the frontal plane; Z, the transverse plane.

## Data Availability

The data presented in this study can be provided according to the requirements of the corresponding authors. The data were not open because of privacy restrictions.
